# Cognitive Functioning and Schizotypy: A Four-Years Study

**DOI:** 10.3389/fpsyt.2020.613015

**Published:** 2021-01-08

**Authors:** Penny Karamaouna, Chrysoula Zouraraki, Stella G. Giakoumaki

**Affiliations:** ^1^Laboratory of Neuropsychology, Department of Psychology, Faculty of Social Sciences, University of Crete, Rethymno, Greece; ^2^University of Crete Research Center for the Humanities, The Social and Educational Sciences (UCRC), University of Crete, Rethymno, Greece

**Keywords:** cognitive functioning, follow-up, schizophrenia-spectrum, schizotypal traits, stability over time

## Abstract

Although there is ample evidence from cross-sectional studies indicating cognitive deficits in high schizotypal individuals that resemble the cognitive profile of schizophrenia-spectrum patients, there is still lack of evidence by longitudinal/follow-up studies. The present study included assessments of schizotypal traits and a wide range of cognitive functions at two time points (baseline and 4-years assessments) in order to examine (a) their stability over time, (b) the predictive value of baseline schizotypy on cognition at follow-up and (c) differences in cognition between the two time points in high negative schizotypal and control individuals. Only high negative schizotypal individuals were compared with controls due to the limited number of participants falling in the other schizotypal groups at follow-up. Seventy participants (mean age: 36.17; 70% females) were assessed at baseline and follow-up. Schizotypal traits were evaluated with the Schizotypal Personality Questionnaire. We found that schizotypal traits decreased over time, except in a sub-group of participants (“schizotypy congruent”) that includes individuals who consistently meet normative criteria of inclusion in either a schizotypal or control group. In these individuals, negative schizotypy and aspects of cognitive-perceptual and disorganized schizotypy remained stable. The stability of cognitive functioning also varied over time: response inhibition, aspects of cued attention switching, set-shifting and phonemic/semantic verbal fluency improved at follow-up. High negative schizotypy at baseline predicted poorer response inhibition and semantic switching at follow-up while high disorganized schizotypy predicted poorer semantic processing and complex processing speed/set-shifting. The between-group analyses revealed that response inhibition, set-shifting and complex processing speed/set-shifting were poorer in negative schizotypals compared with controls at both time points, while maintaining set and semantic switching were poorer only at follow-up. Taken together, the findings show differential stability of the schizotypal traits over time and indicate that different aspects of schizotypy predict a different pattern of neuropsychological task performance during a 4-years time window. These results are of significant use in the formulation of targeted early-intervention strategies for high-risk populations.

## Introduction

Paul Meehl and Gordon Claridge have formulated the two dominant theories in the conceptualization of schizotypy, the quasi-dimensional and the fully-dimensional models, respectively. Meehl (1) proposed that schizotypy refers to a genetically mediated personality profile indicating liability to schizophrenia; when risk-factors for the development of the disease co-occur in individuals with elevated schizotypy, a percentage converts into the disease state. Claridge (2) pays particular attention to individual differences in schizotypal characteristics and considers those as traits lying in a continuum; only when exceeding a critical threshold, schizotypal traits indicate liability to schizophrenia, otherwise remaining part of what he describes as “…*normal individual variation*” [(2), p. 193]. Other reports also quite early suggested that schizotypy indicates proneness to schizophrenia (3) and schizophrenia-spectrum personality disorders (4). Since these early appraisals, (a) aspects of schizotypy have consistently been linked with higher rates of schizophrenia-spectrum traits and/or symptoms (5–7), (b) schizotypal personality has been further established as a key-element of the at risk mental state both in healthy high schizotypal individuals (8) and in individuals at clinical high risk for psychosis (9), and (c) the connection between schizotypy and schizophrenia-spectrum disorders was established over the years at several endophenotypic levels (10–17). Most importantly, high schizotypy has been associated with transition into schizophrenia (18–22).

Schizophrenia is a complex disorder characterized by neuroanatomical (23, 24), genetic (25, 26), psychophysiological (27, 28) and functional (29, 30) impairments. Among these deficits, cognitive decline is a core feature of the disorder as evidenced by neuropsychological (31, 32) as well as functional neuroimaging (33, 34) studies. It is of note that cognitive decline is apparent as early as in the prodromal state (35, 36) and in the first episode (33, 37) of the disorder as well as in individuals at clinical (38, 39) or familial (38, 40) high-risk. To further strengthen the link between schizophrenia and schizotypy, poor cognitive functioning has also been well-established in high schizotypal individuals [for reviews and a meta-analysis see (10, 14, 41)], although the severity of cognitive decline in this population is lower compared with schizophrenia patients (41). Nevertheless, non-significant findings on the relationship between schizotypal traits and cognition have also been reported for several neurocognitive domains, such as set-shifting (42–45), problem solving (46), verbal fluency (46, 47), working memory (48), verbal memory (43, 47–50), processing speed (46, 51), and aspects of attention (50, 52).

In a high percentage of studies, cognitive impairments are reported to remain stable over time in schizophrenic examinees (53, 54). The temporal stability of schizotypy has been demonstrated with confirmatory factor analysis (55). In the same line, schizotypal traits assessed with various versions of the Schizotypal Personality Questionnaire [SPQ; (56)] have been reported (a) to have moderate stability estimates from early adolescence to early adulthood (57, 58), with genetic factors explaining a significant percentage of the reported stability (57), (b) to either decrease, remain stable or increase over a 2 years period depending on their baseline levels in young adults (59), (c) to follow a different trajectory over a 2-years period as negative schizotypy was more stable compared with positive schizotypy in young army conscripts (60) and (d) to remain stable over a 10-years period in psychotic patients and their unaffected siblings, except for disorganized schizotypy which decreased at the 10-years follow up assessment (61). Studies with the Chapman Psychosis Proneness Scales (62–64) in healthy young adults revealed a decrease in physical anhedonia, perceptual aberration and magical thinking at an 18-months (65) and a 2-years follow up assessment (66) compared with baseline along with moderate interclass correlation coefficients of all scores (66). Despite the evidence for stability of schizotypal traits over time, evidence on the association of schizotypy with cognitive functioning in longitudinal or follow-up studies is still limited. To our knowledge, only two studies have thus far dealt with the issue. Thus, (a) Wang et al. (67) reported moderate stability of prospective and working memory deficits in a college sample scoring in the top 10% of the total score in the SPQ over a period of 6 months and (b) Cohen et al. (68) found that the cognitive performance of community participants with increased social anhedonia improved over a 3-years period, except for verbal or visual working memory and attentional vigilance, for which there was no change between the baseline and the follow-up assessments.

In a previous study (69) we examined a wide range of cognitive functions in a large community sample stratified for schizotypal traits, as assessed with the detailed four-factor model of schizotypy (70). The present study aimed to examine (a) the stability of cognitive task performance and schizotypal traits over a 4-years period, (b) the predictive value of baseline schizotypy on cognition after 4 years of the initial assessment of participants and (c) potential differences in cognitive functioning between two time points (i.e., baseline and follow-up assessment after 4 years) in high schizotypal individuals, as defined with the four-factor model (i.e., high paranoid or negative or disorganized or cognitive-perceptual or control individuals). Due to lack of participants fulfilling the criteria in order to be included in any schizotypal group, the latter aim was limited only to the comparison between high negative schizotypals and controls.

## Materials and Methods

### Participants

A total of 263 participants who had taken part in the Prefrontally Mediated Endophenotypes in the Schizophrenia Spectrum (PreMES) study were contacted for the 4-years follow-up assessment. Of those, 193 either declined to participate for several reasons or could not be traced (Supplementary Table 1). Therefore, 70 healthy community participants (mean age ± SD: 36.17 ± 9.38; 21 males/49 females) were assessed both at baseline and follow-up. They were older, had more years of education and comprised fewer females (all *p*-values <0.05) compared with the drop-out group; there were no differences between participants in the follow-up assessment and those who dropped-out in any SPQ measure except for ideas of reference that were slightly higher in the individuals who dropped-out (Supplementary Table 2). Exclusion criteria were as per Karagiannopoulou et al. (69) while a medical history was taken again at follow-up to confirm that there was no health-related change in the participants. The medical history questionnaire was identical at baseline and follow-up assessments and comprised questions related to general health issues [e.g., “Have you visited a medical doctor for any reason during the last five (at baseline)/four (at follow-up) years?” “Are you taking any prescribed or over the counter medication now?” “Are you taking any substances that could be considered addictive, except nicotine and caffeine?”] and questions related specifically to mental health [e.g., “Have you visited a psychiatrist during the last five (at baseline)/four (at follow-up) years?”; Have you visited a psychologist during the last five (at baseline)/four (at follow-up) years, even for counseling required to resolve everyday problems?”; “Do you have any relatives suffering from a psychiatric/psychological disorder?”]; none of the included participants responded positively in any question both at baseline and follow-up. At baseline assessment, participants had been divided into schizotypal groups according to standard criteria (i.e., schizotypy scores falling in the upper 10% for only one schizotypal factor) derived by a normative sample in Greece (70). In detail, (a) negative schizotypals were those with scores ≥18 in the negative schizotypy factor but with scores <14 in the paranoid, <7 in the cognitive-perceptual and <8 in the disorganized schizotypy factors; (b) paranoid schizotypals were those with scores ≥14 in paranoid schizotypy factor but with scores <18 in the negative, <7 in the cognitive-perceptual and <8 in the disorganized schizotypy factors; (c) cognitive-perceptual schizotypals were those with scores ≥7 in the cognitive-perceptual schizotypy factor but with scores <18 in the negative, <14 in the paranoid and <8 in the disorganized schizotypy factors; (d) disorganized schizotypals were those with scores ≥8 in the disorganized schizotypy factor but with scores <18 in the negative, <14 in the paranoid, and <7 in the cognitive-perceptual schizotypy factors; (e) controls were those who did not meet the criteria for any schizotypy factor.

At follow-up, participants were also administered the SPQ (56) and the above-mentioned criteria were applied for their categorization into schizotypal groups a second time. Of the 70 tested volunteers, 54 fell in the same group (31 controls, two cognitive-perceptual, 20 negative, and one disorganized schizotypals—“schizotypy congruent”) and another 16 “converted” into another group (Supplementary Table 3).

The study was approved by the Research Ethics Committee of the University of Crete (approval number: 4/2018/19-03-2018) and the Bureau for the Protection of Personal Data of the Greek State (approval number: ΓN/EΞ/749-1/21-12-2011 and ΓN/EΞ/2029-1/10-11-2016). Following oral presentation of the study's aims and methods, participants received written detailed information and gave written informed consent prior to participation.

### Assessment of Schizotypy

Schizotypal traits were evaluated with the Greek version (70) of the SPQ (56). The SPQ is a 74-dichotomous-item questionnaire and items are grouped into nine subscales (ideas of reference, social anxiety, odd beliefs/magical thinking, unusual perceptual experiences, eccentric/odd behavior, lack of close friends, odd speech, constricted affect, and suspiciousness) in analogy to the diagnostic criteria for Schizotypal Personality Disorder (SPD). The subscales are organized into four schizotypal factors (70): negative (including suspiciousness, social anxiety, lack of close friends and constricted affect), paranoid (including ideas of reference, suspiciousness and social anxiety), cognitive–perceptual (including odd beliefs/magical thinking and unusual perceptual experiences), and disorganized (including eccentric/odd behavior and odd speech).

### Neuropsychological Assessment

#### Wisconsin Card Sorting Test [WCST; (71)]

Set-shifting was assessed with a computerized version of the WCST. The task consisted of four stimulus cards that varied along three dimensions (shape, color, and number) and a target card. Participants were asked to match the target card with one if the stimulus cards and feedback was provided after each selection. In the first part of the task, the first match was always scored as correct and the rule applied by the participant was the first sorting principle. After six consecutive correct responses, the sorting principle changed and participants were informed of this. The next match according to either of the two remaining sorting principles was scored as correct and as previously, after six consecutive correct responses, the sorting principle changed again and participants were informed. The third match was scored as correct only when the last sorting principle was applied. In the second part of the task, participants were required to repeat the three sorting principles in the same order. The task was discontinued when six categories were completed or when the target cards were exhausted. Outcome variables were (a) total number of completed categories, (b) number of unrelated matched cards, (c) perseverative errors either of Milner- or Nelson-type [Milner-type perseverative errors: responses that were correct on the immediately preceding stage of the test (72); Nelson-type perseverative errors: all other perseverative errors (71)], and (d) Milner- and Nelson-non perseverative errors.

#### Letter-Number Sequencing [LNS; (73)]

Executive working memory was examined with the LNS. The Greek version (74) of the task was used. Strings of increasing difficulty comprising intermingled letters and numbers were read to the participants who were required to recite these strings after reordering of the information (i.e., in numeric and alphabetical order). The outcome variable was the total number of correct responses.

#### Trail-Making Test [TMT; (75)]

Processing speed/set-shifting was evaluated with the Greek version (76) of TMT. The task consisted of two parts: in Part A, participants were required to connect 25 consecutively numbered circles, as quickly as possible. In Part B, participants were required to connect 25 consecutively numbered or lettered circles by alternating between the two sequences. The outcome variables were the seconds required by the participants to complete each part of the test.

#### Verbal Fluency Test (77)

The Greek version of the task was used for the assessment of phonemic and semantic fluency. In the phonemic fluency condition, participants were required to produce in 3 consecutive minutes as many as possible words beginning with the letter X (chi), S (sigma), and A (alpha) according to standard instructions. In the semantic fluency condition, participants were required to produce in 3 consecutive minutes, according to standard instructions, as many as possible words belonging to each of the following semantic categories: animals, fruit and objects. Outcome variables were (a) correct responses, (b) perseverative errors (i.e., words generated more than once), (c) intrusion errors (i.e., words that did not belong to the category required), (d) clusters (i.e., three or more consecutive words beginning with the same two letters and having the same sound or two consecutive words that differed only in a vowel sound or words that were homophones for the phonemic part; three or more consecutive words belonging to the same semantic sub-category in the semantic part), and (e) switches (i.e., total correct responses minus the number of words related to each cluster plus number of clusters) per sub-task.

#### Raven's Progressive Matrices (78)

The task assesses abstract reasoning. It comprised five sets of 12 abstract patterns, each with one missing piece. Participants were required to select the choice that best matched the pattern out of the possible answer choices accompanying every pattern. Items within a set were of increasing difficulty. The outcome variable was the total number of correct selections.

#### Cambridge Neuropsychological Test Automated Battery (CANTAB) Tasks (79)

Attention Switch task (AST) assesses cued attentional switching. In every trial, an arrow appears on the left or the right half of the computer screen. A cue presented on the screen indicates whether the participant should make a response about the direction of the arrow or the side of the screen that the arrow was presented. A number of trials includes congruent stimuli (i.e., the arrow is on the right half of the screen and points to the right) whereas another number of trials include incongruent stimuli (i.e., the arrow is on the right half of the screen and points to the left), which require higher cognitive demands. Outcome variables were (a) congruency cost in mean correct responses (i.e., the difference between response latency of congruent vs. incongruent trials), (b) switch cost in mean correct responses (i.e., the difference between response latency of non-switched vs. switched trials), (c) total correct responses in switched and non-switched trials, respectively, (d) total commission errors (i.e., total number of trials in which the examinee responded either before the end of the window or before the appearance of the stimulus) in switched and non-switched trials, respectively.

Stop-Signal task (SST) examines response inhibition. In every trial, a white ring is presented in the center of the screen. After a delay of 500 ms, a white arrow pointing either to the right or to the left is presented within the ring. In the first part of the task, participants were required to press the right-hand button of a touch pad when the arrow points to the right or the left-hand button of the touch pad, when the arrow points to the left. In the second part of the task (five blocks with 64 trials each), participants were required to do as previously unless they hear an auditory stimulus. When they heard the auditory stimulus, participants were required to give no response. Outcome variables were (a) correct responses in the “stop condition” (i.e., when participants are required to give no response), (b) correct responses in the “go condition” (i.e., when participants are required to press either the right- or the left-hand button of the touch pad according to the direction of the arrow), (c) errors in the “stop condition” (i.e., when participants press the button of the touch pad that does not match the direction of the arrow in trials with the auditory stimulus), (d) errors in the “go condition” (i.e., when participants press the button of the touch pad that does not match the direction of the arrow in trials without the auditory stimulus), and (e) reaction time for correct response in the “go condition.”

Stockings of Cambridge [SoC; (80)] examines planning and complex problem solving abilities. Participants were required to compare two different arrangements of “balls” in “socks” (one presented on the top half of the screen, the other at the bottom half) and re-arrange (with the minimum possible number of moves), the balls in the lower half in order to match the target arrangement in the upper half. The problems are of increasing difficulty. Participants were required to plan the complete sequence of moves needed prior to their first move. Outcome variables were (a) number of problems solved correctly with the minimum moves, (b) mean number of moves, (c) mean initial thinking time (i.e., the time taken to organize the solution of the problem prior to execution of the first move), and (d) mean subsequent thinking time (i.e., the time required for the achieving the solution to the problem).

Spatial Working Memory [SWM; (80)] was administered for the assessment of spatial working memory and strategy formation. Participants were required to search through an increasing number of boxes randomly arranged on the screen, until they find a token that, at any one time, is hidden in one of the boxes; upon finding the token, participants were required to place it in a “home area.” The key instruction was that once a token has been found within a particular box, that box should never be used again to hide a token. On each trial, every box is used once to hide one token, such that the total number of tokens to be found equals to the number of boxes on the screen. Outcome variables were (a) between errors (i.e., times of re-visiting a box in which a token was previously found), (b) within errors (i.e., times of re-visiting a box already found to be empty during the same search), (c) double errors (i.e., errors that can be categorized as both within and between errors), (d) strategy score (i.e., an efficient strategy is to follow a predetermined search sequence, beginning with a specified box and then return to start each new sequence with that same box as soon as a token has been found; a high score indicates poor strategy), and (e) mean search preparation time (i.e., the mean time between searches; for the first search it refers to the time between the on-screen presentation of the problem and the first touch of the examinee and for subsequent searches it refers to the time between the placement of token in the home area and the time of the next touch).

### Assessment of Subjective Mood and Feelings on the Day of Testing

Upon arrival at the laboratory, subjects self-rated their mood and feelings on a battery of 16-item visual analog scales [VAS; (81)] of 10 cm each. The raw values for each item were weighted with their respective factor loading and the weighted values were allocated to “alertness,” “anxiety,” and “discontentment” factors (82). The outcome variable was the average of the weighted values for each factor.

### Statistical Analyses

The stability of schizotypal traits and neuropsychological task performance between baseline and follow-up assessments was examined with a series of repeated measures analyses of variance (ANOVAs) with time point (two levels) as the within-subjects factor separately for the total sample (*n* = 70) and the “schizotypy congruent” group (*n* = 54). The rank order stability of participants' schizotypy scores was further examined with Pearson correlations between the baseline and follow-up scores separately for the total sample and the “schizotypy congruent” group. Associations between schizotypal traits at baseline and neuropsychological task performance at follow-up were examined with a series of stepwise regressions (dependent variable: neuropsychological measure; predictors: schizotypal factor scores; confounders: sex, age and smoking habits at baseline) separately for the whole sample and the “schizotypy congruent” group. To reduce the probability of type I error, we applied a Bonferroni correction (0.05/9 neuropsychological tasks = 0.0056); therefore, only *p*-values <0.0056 were considered as significant and *p*-values <0.05 were considered as trends for significance. In the “schizotypy congruent” sub-sample, between-group differences between controls (*n* = 31) and negative schizotypals (*n* = 20) in demographic variables (age, years of education, smoking habits), VAS and SPQ scores at follow-up were examined with parametric or non-parametric tests according to normality of the distribution; sex differences were examined with χ^2^ analysis and differences in neuropsychological tasks performance were examined with 2 × 2 repeated measures ANOVAs with time point (baseline and follow-up) as the within- and group (control or negative schizotypals) as the between-subjects factors. Due to the small sample size of the two groups, we considered the between-group differences as preliminary findings and we did not apply the Bonferroni correction in these analyses.

## Results

### Stability of Schizotypal Traits

In the analyses of the total sample, there was a significant main effect of time point for the cognitive-perceptual [*F*_(1,69)_ = 15.55, *p* < 0.001, η^2^ = 0.184], negative [*F*_(1,69)_ = 6.88, *p* < 0.05, η^2^ = 0.091], paranoid [*F*_(1,69)_ = 14.62, *p* < 0.001, η^2^ = 0.175], and disorganized [*F*_(1,69)_ = 8.22, *p* < 0.005, η^2^ = 0.106] factor scores as well as for the total SPQ score [*F*_(1,69)_ = 19.56, *p* < 0.001, η^2^ = 0.221] and the ideas of reference [*F*_(1,69)_ = 6.41, *p* < 0.05, η^2^ = 0.085], odd beliefs [*F*_(1,69)_ = 14.42, *p* < 0.001, η^2^ = 0.173], unusual perceptual experiences [*F*_(1,69)_ = 4.59, *p* < 0.05, η^2^ = 0.062], eccentric behavior [*F*_(1,69)_ = 4.84, *p* < 0.05, η^2^ = 0.065], odd speech [*F*_(1,69)_ = 7.09, *p* < 0.05, η^2^ = 0.093], and suspiciousness [*F*_(1,69)_ = 9.11, *p* < 0.005, η^2^ = 0.117] sub-scale scores. In all these measures, there was a decrease in the scores at follow-up (Table 1, upper panel). The Pearson's correlations between the factor scores of the two time points revealed that the strongest coefficient was that of negative schizotypy scores (*r* = 0.907, *p* < 0.001) followed by paranoid (*r* = 0.658, *p* < 0.001), cognitive-perceptual (*r* = 0.637, *p* < 0.001), and disorganized (*r* = 0.510, *p* < 0.001) schizotypy. At a sub-scale level, the strongest correlation was found for lack of close friends (*r* = 0.819, *p* < 0.001) followed by strong coefficients for constricted affect (*r* = 0.783, *p* < 0.001), odd speech (*r* = 0.730, *p* < 0.001), excessive social anxiety (*r* = 0.695, *p* < 0.001), eccentric behavior (*r* = 0.625, *p* < 0.001), and suspiciousness (*r* = 0.611, *p* < 0.001) and moderate coefficients for odd beliefs (*r* = 0.589, *p* < 0.001), ideas of reference (*r* = 0.487, *p* < 0.001), and unusual perceptual experiences (*r* = 0.465, *p* < 0.001). The correlation coefficient for the total SPQ score between baseline and follow-up was (*r* = 0.771, *p* < 0.001). A detailed description of the correlation matrix is provided in Supplementary Table 4 (upper panel).

**Table 1:** SPQ metrics (mean ± SD) at baseline and follow-up assessment.

**WHOLE SAMPLE (*N* = 70)**			
	**Baseline assessment**	**Follow-up assessment**	***P*****-value**
Ideas of reference	1.69 ± 1.72	1.21 ± 1.26	**0.014**
Excessive social anxiety	2.80 ± 2.47	2.41 ± 2.12	0.081
Odd beliefs	1.81 ± 1.90	1.09 ± 1.59	** <0.001**
Unusual perceptual experiences	1.11 ± 1.38	0.77 ± 1.19	**0.036**
Eccentric behavior	1.39 ± 1.75	1.00 ± 1.63	**0.031**
Lack of close friends	2.77 ± 2.75	2.73 ± 2.90	0.834
Odd speech	2.43 ± 2.09	1.96 ± 1.92	**0.010**
Constricted affect	2.19 ± 2.16	2.09 ± 2.39	0.582
Suspiciousness	2.40 ± 2.01	1.80 ± 1.71	**0.004**
Total score	18.49 ± 9.98	15.06 ± 9.00	** <0.001**
Cognitive-perceptual factor score	2.93 ± 2.88	1.86 ± 2.33	** <0.001**
Paranoid factor score	6.89 ± 4.14	5.43 ± 3.39	** <0.001**
Negative factor score	9.99 ± 7.01	9.03 ± 7.11	**0.011**
Disorganized factor score	4.03 ± 3.42	2.96 ± 2.81	**0.005**
**SCHIZOTYPY CONGRUENT GROUP (*****N*** **=** **54)**			
Ideas of reference	1.26 ± 1.44	1.13 ± 1.20	0.495
Excessive social anxiety	2.83 ± 2.46	2.54 ± 2.24	0.226
Odd beliefs	1.50 ± 1.65	1.04 ± 1.57	**0.020**
Unusual perceptual experiences	0.78 ± 1.09	0.65 ± 1.01	0.390
Eccentric behavior	1.31 ± 1.75	0.89 ± 1.42	**0.008**
Lack of close friends	3.17 ± 2.96	3.06 ± 3.10	0.640
Odd speech	2.04 ± 1.75	1.78 ± 1.80	0.176
Constricted affect	2.46 ± 2.31	2.41 ± 2.60	0.799
Suspiciousness	2.26 ± 2.08	1.87 ± 1.86	0.100
Total score	17.67 ± 10.37	15.35 ± 9.48	** <0.001**
Cognitive-perceptual factor score	2.27 ± 2.23	1.69 ± 2.06	**0.011**
Paranoid factor score	6.35 ± 3.76	5.54 ± 3.52	**0.039**
Negative factor score	10.50 ± 7.45	9.87 ± 7.66	0.106
Disorganized factor score	3.63 ± 3.34	2.67 ± 2.48	**0.024**

*SPQ, Schizotypal Personality Questionnaire*.

*Significant effects are marked in bold*.

In the analyses of the “schizotypy congruent” group, though, a significant main effect of time point was found only for the cognitive-perceptual [*F*_(1,53)_ = 6.93, *p* < 0.05, η^2^ = 0.116], paranoid [*F*_(1,53)_ = 4.48, *p* < 0.05, η^2^ = 0.078], and disorganized [*F*_(1,53)_ = 5.37, p < 0.05, η^2^ = 0.092] factor scores as well as the total SPQ score [*F*_(1,53)_ = 13.04, *p* < 0.001, η^2^ = 0.198], the odd beliefs [*F*_(1,53)_ = 5.71, *p* < 0.05, η^2^ = 0.097], and eccentric behavior [*F*_(1,53)_ = 7.50, *p* < 0.05, η^2^ = 0.124] sub-scale scores. As previously, the scores at follow-up decreased compared with baseline (Table 1, lower panel). The remaining effects of time point were not significant (all *p*-values >0.100). The Pearson's correlations between the factor scores of the two time points revealed that the strongest coefficient was that of negative schizotypy scores (*r* = 0.931, *p* < 0.001) followed by cognitive-perceptual (*r* = 0.705, *p* < 0.001), paranoid (*r* = 0.700, *p* < 0.001), and disorganized (*r* = 0.482, *p* < 0.001) schizotypy. At a sub-scale level, the strongest correlation was found for lack of close friends (*r* = 0.837, *p* < 0.001) followed by strong coefficients for constricted affect (*r* = 0.796, *p* < 0.001), eccentric behavior (*r* = 0.758, *p* < 0.001), excessive social anxiety (*r* = 0.718, *p* < 0.001), odd speech (*r* = 0.693, *p* < 0.001), suspiciousness (*r* = 0.630, *p* < 0.001), and odd beliefs (*r* = 0.608, *p* < 0.001) and moderate coefficients for ideas of reference (*r* = 0.460, *p* < 0.001) and unusual perceptual experiences (*r* = 0.457, *p* < 0.001). The correlation coefficient for the total SPQ score between baseline and follow-up was *r* = 0.891 (*p* < 0.001). A detailed description of the correlation matrix is provided in Supplementary Table 4 (lower panel).

### Stability of Neuropsychological Task Performance

In the total sample, there was a significant main effect of time point for (a) SST correct responses in the “stop” [*F*_(1,69)_ = 6.28, *p* < 0.05, η^2^ = 0.083] and “go” [*F*_(1,69)_ = 5.79, *p* < 0.05, η^2^ = 0.077] conditions, errors in the “stop” [*F*_(1,69)_ = 12.53, *p* < 0.001, η^2^ = 0.154] and “go” [*F*_(1,69)_ = 6.67, *p* < 0.05, η^2^ = 0.088] conditions and reaction time for correct responses in the “go” condition [*F*_(1,69)_ = 4.01, *p* < 0.05, η^2^ = 0.055], (b) AST correct switched responses [*F*_(1,69)_ = 7.27, *p* < 0.05, η^2^ = 0.097], (c) WCST Milner perseverative errors [*F*_(1,69)_ = 5.05, *p* < 0.05, η^2^ = 0.068] and Nelson non-perseverative-errors [*F*_(1,69)_ = 5.28, *p* < 0.05, η^2^ = 0.071], (d) phonemic fluency correct responses [*F*_(1,69)_ = 17.54, *p* < 0.001, η^2^ = 0.203], perseverative errors [*F*_(1,69)_ = 4.45, *p* < 0.05, η^2^ = 0.061] and switches [*F*_(1,69)_ = 14.84, *p* < 0.001, η^2^ = 0.177], and (e) semantic fluency correct responses [*F*_(1,69)_ = 13.23, *p* < 0.001, η^2^ = 0.161]. In all these measures, performance improved at follow-up compared with baseline (Table 2, upper panel). The remaining effects of time point were not significant (all *p*-values >0.058).

**Table 2:** Neuropsychological task performance (mean ± SD) at baseline and follow-up.

**WHOLE SAMPLE (*N* = 70)**			
	**Baseline assessment**	**Follow-up assessment**	***P*****-value**
**STOCKINGS OF CAMBRIDGE**			
Problems solved correctly	9.37 ± 1.45	9.24 ± 1.42	0.514
Mean moves	4.09 ± 0.36	4.14 ± 0.41	0.277
Mean initial thinking time	5611.63 ± 4316.97	5048.33 ± 2826.96	0.108
Mean subsequent thinking time	416.62 ± 431.95	342.96 ± 374.58	0.116
**STOP-SIGNAL TASK**			
Correct responses—“stop condition”	42.27 ± 5.99	44.17 ± 6.86	**0.015**
Correct responses—“go condition”	238.71 ± 1.48	239.14 ± 1.63	**0.019**
Errors—“stop condition”	0.53 ± 0.74	0.23 ± 0.52	** <0.001**
Errors—“go condition”	1.31 ± 1.46	0.86 ± 1.63	**0.012**
RT correct responses—“go condition”	517.95 ± 124.02	547.21 ± 144.90	**0.049**
**SPATIAL WORKING MEMORY TASK**			
Total between-errors	18.10 ± 14.69	16.87 ± 13.16	0.443
Total within-errors	2.26 ± 3.22	2.51 ± 3.64	0.625
Total double-errors	1.19 ± 2.35	1.06 ± 1.71	0.695
Strategy score	40.97 ± 5.32	41.54 ± 5.82	0.409
Mean search preparation time	1173.79 ± 403.97	1098.17 ± 387.58	0.058
**ATTENTION SWITCH TASK**			
Mean congruency cost—correct responses	81.28 ± 54.22	88.93 ± 63.67	0.377
Mean switch cost—correct responses	−108.32 ± 110.67	−112.16 ± 84.19	0.739
Total correct switched responses	77.33 ± 7.61	79.84 ± 5.20	**0.009**
Total correct non-switched responses	72.30 ± 6.45	72.61 ± 4.57	0.666
Total commission errors—switched responses	0.07 ± 0.31	0.00 ± 0.00	0.058
Total commission errors—non-switched responses	0.03 ± 0.17	0.06 ± 0.24	0.418
**WISCONSIN CARD SORTING TEST**			
Completed categories	5.40 ± 1.12	5.57 ± 0.91	0.187
Unrelated cards	1.19 ± 1.92	0.74 ± 2.51	0.130
Nelson perseverative errors	1.96 ± 2.00	1.57 ± 1.57	0.147
Milner perseverative errors	3.23 ± 2.78	2.34 ± 2.20	**0.028**
Nelson non-perseverative errors	4.60 ± 3.66	3.50 ± 3.06	**0.025**
Milner non-perseverative errors	3.39 ± 3.24	2.73 ± 2.35	0.066
**PHONEMIC VERBAL FLUENCY**			
Correct responses	36.99 ± 8.07	40.33 ± 9.70	** <0.001**
Perseverative errors	0.74 ± 1.15	0.46 ± 0.76	**0.038**
Intrusion errors	1.74 ± 2.21	1.43 ± 1.55	0.300
Clusters	2.16 ± 1.49	2.19 ± 1.89	0.908
Switches	32.07 ± 7.51	35.07 ± 8.39	** <0.001**
**SEMANTIC VERBAL FLUENCY**			
Correct responses	55.07 ± 13.51	60.63 ± 13.35	** <0.001**
Perseverative errors	1.20 ± 1.30	1.01 ± 1.32	0.363
Intrusion errors	3.33 ± 9.23	1.63 ± 4.58	0.077
Clusters	7.93 ± 2.47	8.27 ± 2.69	0.312
Switches	32.61 ± 8.47	34.03 ± 8.25	0.246
**TRAIL MAKING TEST**			
Part A	21.70 ± 6.58	21.07 ± 6.80	0.418
Part B	41.59 ± 11.56	41.01 ± 12.77	0.669
**LETTER-NUMBER SEQUENCING**			
Total correct responses	11.03 ± 3.25	11.20 ± 2.85	0.676
**RAVEN'S PROGRESSIVE MATRICES**			
Total correct responses	52.43 ± 4.73	52.19 ± 5.80	0.585
**SCHIZOTYPY CONGRUENT GROUP (*****N*** **=** **54)**			
	**Baseline assessment**	**Follow-up assessment**	***P*****-value**
**STOCKINGS OF CAMBRIDGE**			
Problems solved correctly	9.39 ± 1.41	9.17 ± 1.42	0.301
Mean moves	4.10 ± 0.38	4.17 ± 0.41	0.239
Mean initial thinking time	5684.15 ± 4399.97	5053.24 ± 3019.83	0.112
Mean subsequent thinking time	390.75 ± 407.65	360.31 ± 391.39	0.569
**STOP-SIGNAL TASK**			
Correct responses—“stop condition”	42.56 **±** 6.16	45.54 **±** 6.37	** <0.001**
Correct responses—“go condition”	238.69 **±** 1.56	239.19 **±** 1.59	**0.012**
Errors—“stop condition”	0.52 **±** 0.75	0.19 **±** 0.52	** <0.001**
Errors—“go condition”	1.35 **±** 1.54	0.81 **±** 1.59	**0.007**
RT correct responses—“go condition”	523.41 **±** 120.82	562.60 **±** 146.02	**0.019**
**SPATIAL WORKING MEMORY TASK**			
Total between-errors	18.22 ± 15.88	17.43 ± 13.90	0.684
Total within-errors	2.44 ± 3.57	2.52 ± 3.70	0.908
Total double-errors	1.30 ± 2.63	1.13 ± 1.83	0.687
Strategy score	40.52 ± 5.76	41.31 ± 5.73	0.320
Mean search preparation time	1186.28 ± 409.65	1122.65 ± 421.64	0.159
**ATTENTION SWITCH TASK**			
Mean congruency cost—correct responses	85.93 ± 56.20	89.69 ± 66.21	0.708
Mean switch cost—correct responses	−104.98 ± 104.69	−108.22 ± 85.79	0.792
Total correct switched responses	78.26 ± 5.85	80.34 ± 5.27	**0.016**
Total correct non-switched responses	72.79 ± 5.26	72.91 ± 4.38	0.886
Total commission errors—switched responses	0.08 ± 0.33	0.00 ± 0.00	0.103
Total commission errors—non-switched responses	0.02 ± 0.14	0.04 ± 0.19	0.569
**WISCONSIN CARD SORTING TEST**			
Completed categories	5.39 ± 1.09	5.56 ± 0.92	0.192
Unrelated cards	1.11 ± 1.70	0.83 ± 2.79	0.384
Nelson perseverative errors	1.91 ± 1.92	1.56 ± 1.59	0.236
Milner perseverative errors	3.07 ± 2.73	2.31 ± 2.20	0.115
Nelson non-perseverative errors	4.50 ± 3.62	3.50 ± 3.18	0.071
Milner non-perseverative errors	3.46 ± 3.46	2.74 ± 2.50	0.092
**PHONEMIC VERBAL FLUENCY**			
Correct responses	36.20 ± 8.35	39.48 ± 9.17	** <0.001**
Perseverative errors	0.76 ± 1.23	0.46 ± 0.77	0.066
Intrusion errors	1.83 ± 2.38	1.33 ± 1.58	0.179
Clusters	2.11 ± 1.63	1.98 ± 1.73	0.634
Switches	31.39 ± 7.69	34.81 ± 8.36	** <0.001**
**SEMANTIC VERBAL FLUENCY**			
Correct responses	55.02 ± 12.67	60.57 ± 12.76	**0.004**
Perseverative errors	1.13 ± 1.26	1.04 ± 1.41	0.684
Intrusion errors	2.81 ± 8.40	0.89 ± 1.99	0.109
Clusters	8.09 ± 2.42	8.48 ± 2.58	0.341
Switches	32.63 ± 8.06	32.94 ± 7.85	0.824
**TRAIL MAKING TEST**			
Part A	21.99 ± 6.33	21.42 ± 7.02	0.522
Part B	42.27 ± 11.19	40.79 ± 10.98	0.291
**LETTER-NUMBER SEQUENCING**			
Total correct responses	10.85 ± 2.93	10.89 ± 2.85	0.965
**RAVEN'S PROGRESSIVE MATRICES**			
Total correct responses	52.30 ± 4.72	52.13 ± 5.67	0.748

*RT, Reaction time*.

*Significant effects are marked in bold*.

In the “schizotypy congruent” group, there was a significant main effect of time point for (a) SST correct responses in the “stop” [*F*_(1,53)_ = 14.08, *p* < 0.001, η^2^ = 0.210] and “go” [*F*_(1,53)_ = 6.78, *p* < 0.05, η^2^ = 0.113] conditions, errors in the “stop” [*F*_(1,53)_ = 14.46, *p* < 0.001, η^2^ = 0.214] and “go” [*F*_(1,53)_ = 7.98, *p* < 0.05, η^2^ = 0.131] conditions and reaction time for correct responses in the “go” condition [*F*_(1,53)_ = 5.83, *p* < 0.05, η^2^ = 0.099], (b) AST correct switched responses [*F*_(1,53)_ = 6.22, *p* < 0.05, η^2^ = 0.107], (d) phonemic fluency correct responses [*F*_(1,53)_ = 13.92, *p* < 0.001, η^2^ = 0.208] and switches [*F*_(1,53)_ = 14.06, *p* < 0.001, η^2^ = 0.210], and (e) semantic fluency correct responses [*F*_(1,53)_ = 8.99, *p* < 0.005, η^2^ = 0.145]. In all these measures, performance improved at follow-up compared with baseline (Table 2, lower panel). The remaining effects of time point were not significant (all *p*-values >0.066).

### Association of Schizotypal Factor Scores at Baseline With Neuropsychological Performance at Follow-Up

#### Stop-Signal Task

In the whole sample, (a) high paranoid schizotypy along with female sex were associated [*F*_(2,69)_ = 7.52, *p* < 0.001, *R*^2^ = 0.183] with fewer correct responses in the “stop condition” (paranoid schizotypy: beta = −0.344, *t* = −3.120, *p* < 0.005; female sex: beta = 0.253, *t* = 2.295, *p* < 0.05), (b) female sex was associated [*F*_(1,69)_ = 13.37, *p* < 0.001, *R*^2^ = 0.164] with more correct responses (beta = 0.405, *t* = 3.657, *p* < 0.001) in the “go condition,” (c) high negative schizotypy was associated [*F*_(1,69)_ = 5.78, *p* < 0.005, *R*^2^ = 0.147] with more errors in the “stop condition” (beta = 0.293, *t* = 2.535, *p* < 0.05). In the “schizotypy congruent” sub-sample, only high negative schizotypy was associated [*F*_(1,53)_ = 7.85, *p* < 0.001, *R*^2^ = 0.235] with errors in the “stop condition” (beta = 0.392, *t* = 3.113, *p* < 0.005).

#### Spatial Working Memory

In the whole sample, only older age was significantly associated [*F*_(1,69)_ = 9.02, *p* < 0.005, *R*^2^ = 0.117] with more total between errors (beta = 0.342, *t* = 3.003, *p* < 0.005) and prolonged mean search preparation time [*F*_(1,69)_ = 9.50, *p* < 0.005, *R*^2^ = 0.123; beta = 0.350, *t* = 3.082, *p* < 0.005]. In the “schizotypy congruent” group, a similar pattern was observed: older age was significantly associated [*F*_(1,53)_ = 9.93, *p* < 0.005, *R*^2^ = 0.160] with more total between errors (beta = 0.400, *t* = 3.150, *p* < 0.005) and longer mean search preparation time [*F*_(1,53)_ = 11.85, *p* < 0.001, *R*^2^ = 0.186; beta = 0.431, *t* = 3.442, *p* < 0.001].

#### Wisconsin Card Sorting Test

In the whole sample, only high negative schizotypy tended to be associated [*F*_(1,69)_ = 5.31, *p* < 0.05, *R*^2^ = 0.072] with fewer completed categories (beta = −0.269, *t* = −2.303, *p* < 0.05). In the “schizotypy congruent” group, we did not find any significant models (all *p*-values >0.05).

#### Phonemic Verbal Fluency

In the whole sample, only older age tended to be associated [*F*_(1,69)_ = 5.95, *p* < 0.05, *R*^2^ = 0.284] with more perseverative errors (beta = 0.284, *t* = 2.439, *p* < 0.05). In the “schizotypy congruent” sub-sample, female sex was (a) associated [*F*_(1,53)_ = 8.69, *p* < 0.005, *R*^2^ = 0.143] with correct responses (beta = 0.378, *t* = 2.948, *p* < 0.005) and (b) associated [*F*_(1,53)_ = 9.20, *p* < 0.005, *R*^2^ = 0.150] with more clusters (beta = 0.388, *t* = 3.033, *p* < 0.005).

#### Semantic Verbal Fluency

In the whole sample, (a) high disorganized schizotypy along with more cigarettes smoked daily were associated [*F*_(2,69)_ = 7.40, *p* < 0.001, *R*^2^ = 0.181] with more intrusion errors (disorganized schizotypy: beta = 0.329, *t* = 2.959, *p* < 0.005; cigarettes: beta = 0.239, *t* = 2.147, *p* < 0.05) and (b) high negative schizotypy score was associated [*F*_(1,69)_ = 9.92, *p* < 0.005, *R*^2^ = 0.127] with fewer switches (beta = −0.357, *t* = −3.149, *p* < 0.005). High disorganized schizotypy tended to be associated [*F*_(1,69)_ = 4.03, *p* < 0.05, *R*^2^ = 0.056] with fewer correct responses (beta = −0.236, *t* = −2.007, *p* < 0.05). In the “schizotypy congruent” group, high negative schizotypy was associated [*F*_(1,53)_ = 12.32, *p* < 0.001, *R*^2^ = 0.192] with fewer switches (beta = −0.438, *t* = −3.510, *p* < 0.001) and tended to be associated [*F*_(1,53)_ = 6.14, *p* < 0.05, *R*^2^ = 0.106] with more intrusion errors (beta = 0.325, *t* = 2.477, *p* < 0.05).

#### Trail Making Test

In the whole sample, (a) older age was associated [*F*_(1,69)_ = 9.38, *p* < 0.005, *R*^2^ = 0.121] with prolonged completion time of the first part (beta = 0.348, *t* = 3.062, *p* < 0.005) and (b) high disorganized schizotypy along with high cognitive-perceptual schizotypy and more cigarettes smoked daily were associated [*F*_(3,69)_ = 11.25, *p* < 0.001, *R*^2^ = 0.338] with prolonged completion time of the second part of the task (disorganized schizotypy: beta = 0.381, *t* = 3.721, *p* < 0.001; cognitive-perceptual schizotypy: beta = 0.215, *t* = 2.083, *p* < 0.05; cigarettes: beta = 0.339, *t* = 3.325, *p* < 0.001). In the “schizotypy congruent” sub-group, (a) older age was again associated [*F*_(1,53)_ = 9.09, *p* < 0.005, *R*^2^ = 0.149] with prolonged completion time of the first part (beta = 0.386, *t* = 3.016, *p* < 0.005) and (b) only high disorganized schizotypy was associated [*F*_(1,53)_ = 18.60, *p* < 0.001, *R*^2^ = 0.263] with prolonged completion time of the second part of the task (beta = 0.513, *t* = 4.312, *p* < 0.001).

### Differences Between Controls and Negative Schizotypals

#### Demographics, VAS, and SPQ Scores

There were no differences in any demographic variables or VAS scores between the control and the negative schizotypal groups (all *p*-values >0.07). The negative schizotypal group, though, had higher total SPQ, paranoid, negative and disorganized factor scores (all *p*-values <0.005). A detailed description is provided in Table 3.

**Table 3:** Demographic characteristics, VAS, and SPQ scores (mean ± SD) of the control and negative schizotypal groups.

	**Controls** **(*n* = 31)**	**Negative schizotyplas (*n* = 20)**	***P*-value**
Age (years)^a^	41.32 ± 10.22	38.80 ± 7.52	0.347
Education (years)^a^	17.27 ± 2.05	16.05 ± 2.63	0.072
Sex (male/female)^b^	7/24	9/11	0.092
Cigarettes/day^c^	3.74 ± 7.03	3.10 ± 7.30	0.250
VAS anxiety^a^	2.49 ± 1.79	2.47 ± 1.52	0.969
VAS discontentment^a^	1.48 ± 1.01	2.00 ± 1.01	0.083
VAS alertness^a^	5.30 ± 0.75	5.10 ± 1.03	0.437
SPQ total score^a^	8.26 ± 4.95	25.65 ± 3.67	** <0.001**
SPQ Cognitive-Perceptual factor score^c^	1.42 ± 1.93	1.50 ± 1.50	0.385
SPQ Paranoid factor score^a^	3.81 ± 2.83	8.10 ± 3.14	** <0.001**
SPQ Negative factor score^c^	4.13 ± 3.05	19.20 ± 1.06	** <0.001**
SPQ Disorganized factor score^c^	1.61 ± 1.36	3.90 ± 2.63	**0.003**

a *One-way Analysis of variance*.

b *Chi-square analysis*.

c *Mann-Whitney analysis*.

*SPQ, Schizotypal Personality Questionnaire*.

*VAS, Visual Analog Scales*.

*Significant between-group differences are marked in bold*.

#### Neuropsychological Task Performance

The descriptives of the two groups' performance in the tasks with either between-group differences or interactions involving group are presented in Table 4; the descriptives of the remaining tasks are presented in Supplementary Table 5.

**Table 4:** Neuropsychological task performance (mean ± SD) of the control and negative schizotypal groups.

	**Controls (*****n*** **=** **31)**		**Negative schizotypals (*****n*** **=** **20)**		***P*-value group**	***P*-value time point**	***P*-value group × time point**
	**Baseline**	**Follow-up**	**Baseline**	**Follow-up**			
**STOP-SIGNAL TASK**							
Correct responses—“stop condition”	44.45 ± 6.16	47.10 ± 5.84	39.80 ± 5.32	43.70 ± 6.33	**0.009**	** <0.001**	0.464
Correct responses—“go condition”	239.19 ± 0.98	239.55 ± 1.09	237.90 ± 1.92	238.85 ± 1.66	**0.006**	**0.001**	0.128
Errors—“stop condition”	0.39 ± 0.62	0.00 ± 0.00	0.70 ± 0.86	0.45 ± 0.76	**0.013**	**0.001**	0.470
Errors—“go condition”	0.90 ± 1.04	0.45 ± 1.09	2.05 ± 1.88	1.15 ± 1.66	**0.011**	**0.001**	0.248
RT correct responses—“go condition”	558.60 ± 112.09	590.48 ± 137.57	469.39 ± 123.20	530.10 ± 158.33	**0.031**	**0.011**	0.413
**WISCONSIN CARD SORTING TEST**							
Completed categories	5.68 ± 0.65	5.81 ± 0.48	4.85 ± 1.46	5.25 ± 1.21	**0.007**	**0.032**	0.264
Unrelated cards	1.13 ± 1.28	0.26 ± 0.51	1.20 ± 2.31	1.85 ± 4.43	0.159	0.739	**0.025**
Nelson perseverative errors	1.87 ± 1.48	1.65 ± 1.43	1.85 ± 2.54	1.30 ± 1.78	0.654	0.214	0.601
Milner perseverative errors	3.03 ± 2.56	2.68 ± 2.29	2.90 ± 2.99	1.70 ± 1.98	0.273	0.127	0.403
Nelson non-perseverative errors	4.81 ± 3.47	3.65 ± 2.82	4.25 ± 4.04	3.15 ± 3.57	0.513	0.055	0.958
Milner non-perseverative errors	3.65 ± 2.58	2.61 ± 1.84	3.50 ± 4.68	2.75 ± 3.26	0.996	0.046	0.747
**SEMANTIC VERBAL FLUENCY**							
Correct responses	54.00 ± 10.74	62.23 ± 12.60	56.60 ± 15.05	59.75 ± 12.83	0.984	**0.005**	0.195
Perseverative errors	1.13 ± 1.09	1.16 ± 1.46	1.15 ± 1.57	0.65 ± 0.81	0.394	0.310	0.249
Intrusion errors	2.35 ± 7.47	0.42 ± 0.72	3.90 ± 10.30	1.65 ± 3.01	0.276	0.112	0.904
Clusters	8.00 ± 2.14	8.48 ± 2.84	8.15 ± 2.91	8.50 ± 2.35	0.889	0.349	0.880
Switches	32.74 ± 7.39	36.06 ± 7.81	33.10 ± 9.29	29.20 ± 5.89	0.055	0.843	**0.016**
**TRAIL MAKING TEST**							
Part A	22.85 ± 6.86	22.44 ± 8.20	20.32 ± 5.27	19.30 ± 4.75	0.095	0.458	0.754
Part B	40.39 ± 9.07	36.54 ± 8.26	45.45 ± 13.56	45.03 ± 10.56	**0.012**	0.116	0.206

*Significant effects are marked in bold*.

*RT, Reaction Time*.

##### Stop Signal Task

Significant main effects of group and time point were found for the correct responses in the “stop” [group: *F*_(1,49)_ = 7.43, *p* < 0.01; η^2^ = 0.132; time point: *F*_(1,49)_ = 14.81, *p* < 0.001; η^2^ = 0.232] and “go”[group: *F*_(1,49)_ = 8.27, *p* < 0.01; η^2^ = 0.144; time point: *F*_(1,49)_ = 11.52, *p* < 0.001; η^2^ = 0.190] conditions, in the errors made in the “stop” [group: *F*_(1,49)_ = 6.68, *p* < 0.05; η^2^ = 0.120; time point: *F*_(1,49)_ = 11.46, *p* < 0.001; η^2^ = 0.189] and “go” [group: *F*_(1,49)_ = 7.04, *p* < 0.05; η^2^ = 0.126; time point: *F*_(1,49)_ = 12.44, *p* < 0.001; η^2^ = 0.202] conditions and in the reaction time for correct responses [group: *F*_(1,49)_ = 4.95, *p* < 0.05; η^2^ = 0.092; time point: *F*_(1,49)_ = 7.04, *p* < 0.05; η^2^ = 0.126] in the “go” condition. Overall, (a) the negative schizotypal group gave fewer correct responses, made more errors in both conditions and had prolonged reaction time in the correct responses of the “go” condition compared with the control group and (b) both groups gave more correct responses, made fewer errors and had longer reaction time at follow-up compared with the baseline assessment.

##### Wisconsin Card Sorting Test

**Figure 1:**
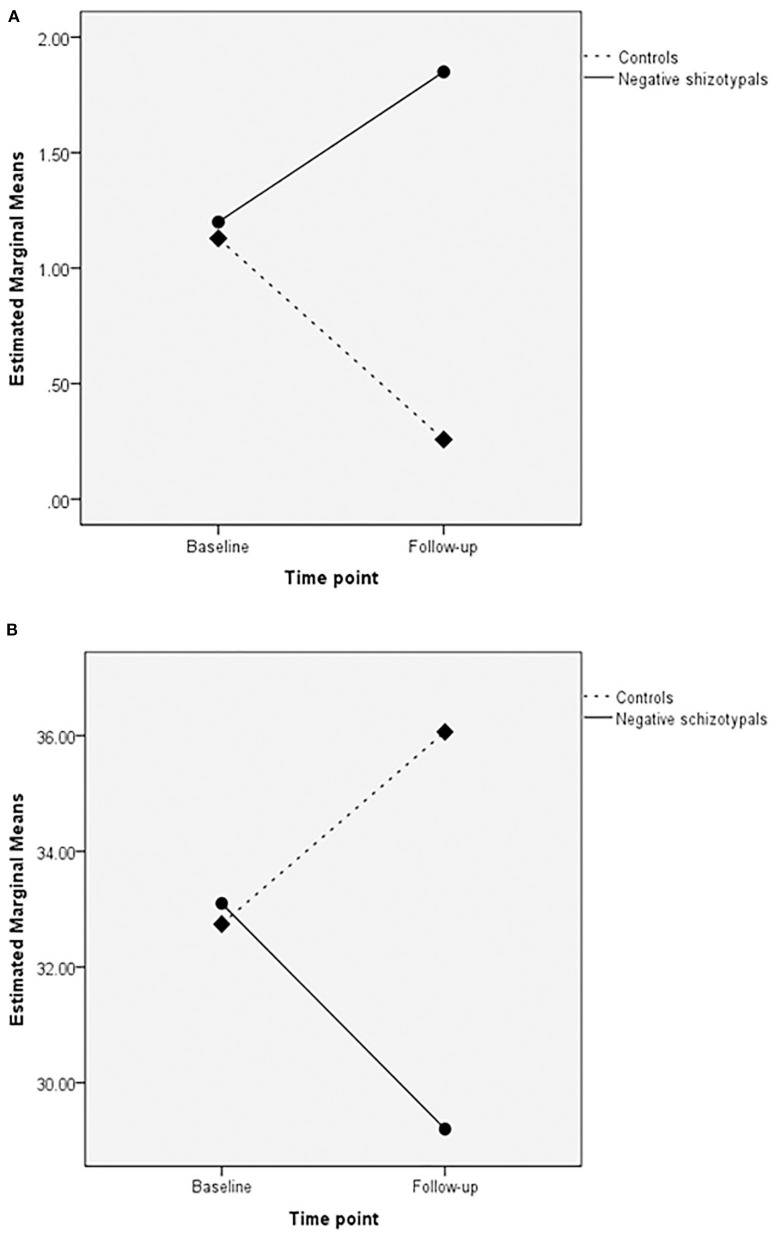
Group × time point interactions in the unrelated cards of the Wisconsin Card Sorting test (upper panel; **A**) and switches in semantic verbal fluency (lower panel; **B**).

The negative schizotypal group completed fewer categories [main effect of group: *F*_(1,49)_ = 8.06, *p* < 0.01; η^2^ = 0.141] compared with the control group. Both groups completed more categories and made fewer Milner non-perseverative errors at follow-up compared with the baseline assessment [main effect of time point for completed categories: *F*_(1,49)_ = 4.86, *p* < 0.05; η^2^ = 0.090; main effect of time point for Milner non-perseverative errors: *F*_(1,49)_ = 4.20, *p* < 0.05; η^2^ = 0.079]. We also found a significant group × time point interaction for unrelated cards [*F*_(1,49)_ = 5.34, *p* < 0.05; η^2^ = 0.098]; the negative schizotypal group selected more unrelated cards at follow-up compared with the baseline assessment, while the opposite pattern was observed in the control group (Figure 1, upper panel). No other significant main effects or interactions were found (all *p*-values >0.055).

##### Trail Making Test

The negative schizotypal group had poorer performance in the second part of the task at both time points [main effect of group: *F*_(1,49)_ = 6.83, *p* < 0.05; η^2^ = 0.122]. We did not find any other significant main effects or interactions (all *p*-values >0.095].

##### Phonemic Verbal Fluency

Significant main effects of time point were found for correct responses [*F*_(1,49)_ = 10.76, *p* < 0.005; η^2^ = 0.180] and number of switches [*F*_(1,49)_ = 13.14, *p* < 0.001; η^2^ = 0.211] as both groups gave more correct responses and made more switches at follow-up compared with the baseline assessment. No other significant main effects or interactions were revealed (all *p*-values >0.062).

##### Semantic Verbal Fluency

A significant group × time point interaction was revealed for the number of switches [*F*_(1,49)_ = 6.20, *p* < 0.05; η^2^ = 0.112] according to which, the negative schizotypal group made fewer switches at follow-up compared with the baseline assessment, while the control group presented with the opposite pattern (Figure 1, lower panel). We also found a significant main effect of time point for the number of correct responses [*F*_(1,49)_ = 8.67, *p* < 0.005; η^2^ = 0.150] with both groups improving at follow-up. The analyses did not reveal any other significant main effects or interactions (all *p*-values >0.055).

##### Attention Switch Task

A significant main effect of time point was revealed for the total correct switched responses [*F*_(1,49)_ = 6.42, *p* < 0.05; η^2^ = 0.118], with both groups improving at follow-up. The remaining main effects or interactions were not significant (all *p*-values >0.106).

## Discussion

### Stability of Schizotypal Traits and Neuropsychological Tasks Performance

As regards schizotypal traits, a different pattern emerged when examining the total sample and the sub-sample of “schizotypy congruent” individuals (i.e., participants who at follow-up fulfilled the criteria of inclusion in the same schizotypal group as in the baseline assessment) separately. Thus, in the total sample we found that schizotypal traits did not remain stable over a 4-years period, as a decrease in the scores of all schizotypal factors, SPQ total score and the majority of sub-scale scores was found at follow-up. Contrary to this, in the “schizotypy congruent” sub-group, negative schizotypy (and all its constituents) remained unchanged, as did ideas of reference (which play a central role in paranoid schizotypy), unusual perceptual experiences (which is a core feature of cognitive-perceptual schizotypy) and odd speech (one of the two characteristics of disorganized schizotypy). The decrease in schizotypal traits at follow-up is in accordance with findings indicating fluctuations in SPD-like features (59, 83, 84) or schizotypal traits *per se* (61, 65, 66) over time. Nevertheless, certain schizotypal traits (i.e., the negative schizotypal dimension, ideas of reference, unusual perceptual experiences and odd speech) seem to have a more enduring nature in certain individuals (i.e., those falling in the extreme ends in the continuum of schizotypy). Plausible explanations for these findings are based on the facts that negative schizotypy is the sub-clinical analog of negative symptoms, which have been reported to be a stable feature of schizophrenia (85, 86). Further supporting the association of negative schizotypy with negative symptoms, Cohen et al. (84) recently reported that individuals with increased social anhedonia presented with increased negative symptom characteristics over a 3-years period. With respect to the other schizotypal traits that remained stable, unusual perceptual experiences and ideas of reference have also been reported to show stability over time in SPD (87) as is the case with odd/disorganized speech in schizophrenia (88). The correlational analyses aimed to further explore the rank order stability of participants' scores, i.e., “…*how individuals maintain their standing on a trait level compared to others in a population over time*” [(89), p. 27]. We found that the rank order stability of negative, paranoid and cognitive-perceptual factor scores ranged from very high to high while disorganized schizotypy was moderately rank-ordered; both groups also had a similar pattern of rank order stability at a sub-scale level. This is the first study to report findings on the rank order stability of schizotypal traits with the four-factor model and it is especially interesting that the reported findings are in accordance with the schizophrenia literature indicating high rank order stability of negative symptoms (90) as well as findings suggesting that aspects of paranoid and cognitive-perceptual schizotypy are the most prevalent and highly rank ordered features of SPD (87).

In accordance with previous studies that have employed the same neuropsychological tasks as in the present one and have examined the stability of cognitive functioning (91–96), we found that there was no difference in planning/problem solving, spatial working memory/strategy formation, processing speed, executive working memory and abstract reasoning between the two time points. On the other hand, response inhibition and aspects of cued attention switching, set-shifting as well as phonemic and semantic verbal fluency improved at follow-up compared with baseline, although the effect of time on set-shifting was abolished in the “schizotypy congruent” sub-group probably due to the smaller sample size. Even though there are findings supporting the stability of these cognitive functions over time (93, 97–99), there is also evidence indicating that performance in the tasks employed here is subject to practice effects resulting in improved performance (99–103). The majority of these studies include assessments at shorter time-intervals than in the present one; however, there are findings supporting the persistence of these effects at time intervals comparable to the 4-years of our follow-up assessment (104–106).

### Prediction of Neuropsychological Performance at Follow-Up by Baseline Schizotypy

This is the first study to explore the predictive significance of schizotypal traits on cognitive functions after a 4-years period. Moreover, we examined the afore-mentioned associations in our total sample and in the sub-sample of “schizotypy congruent” individuals, separately. It was interesting to find out that a different pattern of associations was revealed between different schizotypal dimensions or specific schizotypal traits in the two groups.

High negative schizotypy at baseline predicted poorer response inhibition (as examined with more errors in the most “inhibition-demanding” condition of the SST task) and poorer semantic switching (i.e., shifting to another semantic category in order to produce more correct responses) 4 years later both in the total sample and in the “schizotypy congruent” group. Ettinger et al. (107) have reported that negative schizotypy is associated with poor response inhibition cross-sectionally, in analogy to findings showing associations between negative schizophrenia symptoms with both response inhibition (108) and semantic switching (109). The present findings, therefore, suggest that the negative effect of schizotypy on these two executive processes also remains stable over time. Response inhibition and semantic switching are mediated by a frontal-temporal-parietal network in healthy individuals (110–113) and in schizophrenia patients (114–116). Interestingly, alterations within this network seem to be of central importance in SPD as reduced gray matter volume (117, 118) has been reported in SPD patients along with associations between cortical thinning (119) or gray matter volume reductions (118) with SPD symptoms. Although the literature on schizotypy is still limited, there is evidence implicating this neural network in negative schizotypy (120, 121), as well. Therefore, the critical link between all implicated constructs (i.e., negative schizotypy, response inhibition, semantic switching, schizophrenia, SPD) seems to be the neural circuitry connecting the frontal, temporal, and parietal lobes. Although the methodology of the present study allows only for indirect conclusions, we propose that inefficient functioning of/processing within this network is a persistent feature of negative schizotypy identifying sub-optimal cognitive functioning mediated by this network over time.

High disorganized schizotypy at baseline predicted more intrusion errors and tended to predict fewer correct responses in the semantic fluency task only in the total sample. Tan and Rossell (122) have already reported reduced semantic fluency productivity as disorganized schizotypy increases. In the present study, though, we found a stronger predictive value of disorganized schizotypy for semantic intrusion errors (i.e., production of words belonging to a semantically different category). Correct responses potentially reflect semantic information reserve capacities, while semantic intrusion errors could be considered as failures in semantic processing either due to poor strategic search of representations classified according to their meaning or misattribution of meaning to representations. It is of note, that disorganized schizotypy has been associated with a wide neural network encompassing several brain structures, such as the superior longitudinal fasciculus (121), hippocampus (123), and superior temporal gyrus (124), that are crucial for semantic processing (125–131), thus proving a plausible explanation for our finding. The fact that the association of disorganized schizotypy and semantic processing was abolished in the “schizotypy congruent” sub-group is most probably to the smaller sample size of this group resulting in limited variation of semantic fluency intrusion errors that is required for associational analyses.

High disorganized schizotypy at baseline also predicted poorer complex processing speed/set-shifting, as assessed with the multitasking second part of TMT, both in the total sample and “schizotypy congruent” individuals. In support of this finding, cross-sectional studies have revealed that disorganized schizotypy correlates with response inhibition in relatives of schizophrenia patients as well as controls (107) and disorganization symptoms in schizophrenia patients have been associated with TMT performance (132, 133) and other measures of response inhibition (134). At a neuroanatomical level, the neural substrate underlying performance in TMT includes a wide frontal-temporal-parietal network with a frontal cluster including the cingulate and insular cortices and the frontal gyrus acting as a central node (135). Disorganized schizotypy has been associated with reduced volume of the insula (124), cingulate cortex and frontal gyrus (121, 124) as well as altered insular information processing (136), and thinning of the anterior cingulate (137). Taken together, our findings suggest that the association of disorganized schizotypy with cognitive functioning relies on different mechanisms and holds out over time: a frontal-temporal network mediates the relationship of disorganized schizotypy with semantic processing while a cluster of frontal regions is the key-neural substratum for connecting disorganized schizotypy with complex processing speed/set-shifting.

### Differences in Neuropsychological Tasks Performance Between Negative Schizotypals and Controls

The follow-up assessment did not include participants falling in all schizotypal dimensions due to the high-rate of drop-out; thus, between-group differences were limited between negative schizotypals and controls and due the between-group differences in most schizotypal dimensions, we could assume that the former group has an overall “heavier dose” of schizotypal traits compared with the latter. As regards the effects of time and in accordance with the analyses of the total sample, neuropsychological task performance in both groups improved at follow-up in measures of response inhibition, set-shifting, phonemic and semantic fluency and cued attention switching. The analyses also revealed that the group of negative schizotypals had poorer response inhibition (as assessed with the SST), set-shifting (as assessed with the completed categories in the WCST), and complex processing speed/set-shifting (examined with the second part of TMT) compared with controls at both time points. These findings (a) cannot be attributed to differences in the demographic characteristics of the participants as there were no between-group differences in these variables, (b) are in accordance with previous cross-sectional studies (69, 107, 138), (c) highlight the persistent nature of specific inefficiencies in cognition in negative schizotypy, and (d) supplement the association of this schizotypal dimension with processes underlaid by the frontal-temporal-parietal network described in the previous section.

Interesting group × time point interactions were also revealed in two fundamental measures: the negative schizotypal group was more prone to failure to maintain set (139) as indicated by the selection of more unrelated cards in the WCST (i.e., choosing a card that has no common features with the target stimuli after acquiring the sorting principle) and made fewer semantic switches at follow-up compared with baseline performance; the opposite pattern was observed in controls. Maintaining set and switching are inter-related as they both require efficient self-monitoring, response inhibition and vigilance. They are also primary features of successful performance in the WCST and semantic fluency tasks [e.g., reported correlations between failure to maintain set and completed categories as well as perseverative errors (140) and between semantic switching and word production (141, 142)]. With respect to the present findings, it is tempting to propose that maintaining set and semantic switching in negative schizotypals are progressively deteriorating thus leading to increased effort for the successful completion of the tasks. These findings, however, are preliminary and the aforementioned suggestion should be viewed with greater caution as far as maintaining set is concerned due to the reported low test-retest reliability of this measure (143, 144).

### Conclusions

Taken together, the findings of the present study indicate that schizotypal traits, when analyzed with a detailed four-factor model, decrease over a 4-years period in the general population. The exception is negative schizotypy and odd speech, which is a central feature of disorganized schizotypy; these traits remained stable, in accordance to the literature on schizophrenia and spectrum disorders. The stability of cognitive functioning also varied over time. Thus, response inhibition, aspects of cued attention switching, set-shifting as well as phonemic and semantic verbal fluency are more likely to improve, possibly due to persistent learning/practice effects when performing the same test twice. The need for alternate forms of neuropsychological tasks has already been highlighted (145, 146) and the present study suggests that this applies not only in clinical but also in research settings.

High negative schizotypy at baseline predicted poorer response inhibition and semantic switching at follow-up, further supporting and supplementing the involvement of a frontal-temporal-parietal network in all these latent constructs. High disorganized schizotypy at baseline predicted poorer semantic processing and complex processing speed/set-shifting. A frontal-temporal network is suggested to mediate the association of disorganized schizotypy with semantic processing while a cluster of frontal regions is suggested as the key-element for its connection with complex processing speed/set-shifting. The association of negative schizotypy with cognition over time was further explored with preliminary findings on differences between negative schizotypal and control individuals. Poor response inhibition, set-shifting, and complex processing speed/set-shifting were found to be consistently impaired in the former group, as they performed lower than controls in both time points. The ability to maintain set and semantic switching, though, were found to be progressively deteriorating in negative schizotypy. The present findings highlight the importance of taking into consideration the differences in schizotypal traits when designing early-intervention programs for high-risk populations. The fact that different association patterns with cognition were revealed, further advances the formulation of more targeted approaches depending on the prevailing schizotypal traits not only in the schizophrenia spectrum but also in other clusters of mental disorders; for example features of negative schizotypy have been associated with aspects (147–149) or the prevalence rates (5) of mood disorders.

### Strengths and Limitations of the Study

To our knowledge, this is the first study exploring associations of schizotypal traits with cognitive functioning over a 4-years period. It is of note that the participants were community residents covering a wide age-range instead of the most commonly included college students' samples that have been reported to under-represent the general population (150). For the assessment of schizotypy we applied the detailed four-factor model, which allows for more thorough delineations of schizotypal traits. We also applied strict normative criteria for the identification of negative schizotypal and control individuals instead of dividing the participants according to a sample-wise approach (e.g., by median or percentile splits in the current sample).

Nevertheless, there are certain limitations that should be taken into account. First, we had a quite high drop-out rate in the initial sample resulting in a small sample size at follow-up that also differed in demographics compared with the drop-out participants. This also resulted in a small number of “schizotypy incongruent” individuals that were not examined at all at follow-up. Second, we assessed schizotypy only with a self-report scale, which is widely used but was recently reported not to be fully concordant with interview-based assessments (151). Third, although we examined the subjective state of mood and feelings on the day of testing, we did not include these data in our regression analyses (this would increase the complexity of our models to a level that could not be justified by the current sample sizes) and we did not examine other factors (e.g., anxiety or discontentment on a daily basis) that might have interfered with the participants' performance, especially since the study was conducted during the COVID-19 pandemic.

## Data Availability Statement

The raw data supporting the conclusions of this article will be made available by the authors, without undue reservation.

## Ethics Statement

The studies involving human participants were reviewed and approved by the Research Ethics Committee of the University of Crete. The patients/participants provided their written informed consent to participate in this study.

## Author Contributions

PK collected the data, conducted the initial analyses, and wrote the first draft of the manuscript. CZ collected the data and supplemented the manuscript. SG designed the study and supplemented the statistical analyses and the manuscript. All authors contributed to the article and approved the submitted version.

## Conflict of Interest

The authors declare that the research was conducted in the absence of any commercial or financial relationships that could be construed as a potential conflict of interest.
